# Synchronous epithelioid sarcoma of bilateral thigh: a rare case report and literature review

**DOI:** 10.3389/fonc.2026.1803611

**Published:** 2026-03-31

**Authors:** Jia Li, Sicheng Wang, Mingfeng Zhong, Hanyang Yu

**Affiliations:** 1 Department of Ultrasound, Weiyuan County People’s Hospital, Neijiang, Sichuan, China; 2 College of Physical Education and Health, Sichuan Institute of Industrial Technology, Deyang, Sichuan, China; 3 Department of Urology, Weiyuan County People’s Hospital, Neijiang, Sichuan, China

**Keywords:** case report, epithelioid sarcoma, imaging, review, synchronous

## Abstract

Epithelioid sarcoma(ES) is an exceedingly rare soft-tissue sarcoma, accounting for approximately 1% of all soft-tissue sarcomas. Despite its typically indolent clinical course, ES carries a substantial risk of local recurrence and distant metastasis, underscoring the importance of early recognition. However, diagnosis is often delayed because clinical suspicion is low and ultrasonography(US)/magnetic resonance imaging(MRI) are frequently nonspecific. In this report, a 47-years-old woman was admitted after incidentally noticing egg-sized masses on the medial aspects of both mid-thighs for one week. Lower Extremity US and Lower Extremity MRI both failed to correctly diagnose the bilateral thigh tumors. Histopathological examination demonstrated malignant tumors arising from skeletal muscle in both thighs. Immunophenotypic findings confirmed a diagnosis of classic-type ES. We further review the literature to summarize the clinical and US/MRI characteristics of ES and to highlight diagnostic pitfalls and current therapeutic strategies.

## Introduction

Epithelioid sarcoma(ES) is a rare malignant soft-tissue neoplasm of mesenchymal origin with uncertain histogenesis, accounting for approximately 1% of all soft-tissue sarcomas, first described by Enzinger in 1970 (1). ES is characterized by the loss of expression of *SMARCB1*, a key member of the SWItch/Sucrose Non-Fermentable (SWI/SNF) chromatin remodeling complex (2). It predominantly affects young adults and exhibits a slight male predominance. Although ES generally demonstrates relatively slow tumor growth, it is biologically aggressive and carries a high risk of local recurrence and distant metastasis (3, 4). Because of its infiltrative growth pattern and nonspecific clinical and radiologic manifestations, ES is frequently misdiagnosed as a benign or inflammatory lesion, which often leads to delayed diagnosis and treatment (5). As a result, early recognition and accurate diagnosis remain major challenges in both clinical practice and imaging evaluation. In this report, a 47-years-old woman was admitted after incidentally noticing egg-sized masses on the medial aspects of both mid-thighs for one week. Lower Extremity ultrasonography(US) and Lower Extremity magnetic resonance imaging(MRI) both failed to correctly diagnose the bilateral thigh tumors. Histopathological examination demonstrated malignant tumors arising from skeletal muscle in both thighs. Immunophenotypic findings confirmed a diagnosis of classic-type ES. With a review of the literature, we summarize its clinical and imaging characteristics to enhance awareness and recognition of this rare entity.

## Case description

A 47-years-old woman was admitted after incidentally noticing egg-sized masses on the medial aspects of both mid-thighs for one week. She reported no associated pain or progressive enlargement of the lesions and had no relevant medical history or history of trauma. On physical examination, localized bulging was observed on the medial mid-thighs bilaterally, with normal skin temperature and no erythema or tenderness. Palpation revealed firm masses with poorly defined margins. Lower-limb range of motion was preserved, and distal perfusion and sensation were intact.

**Figure 1:**
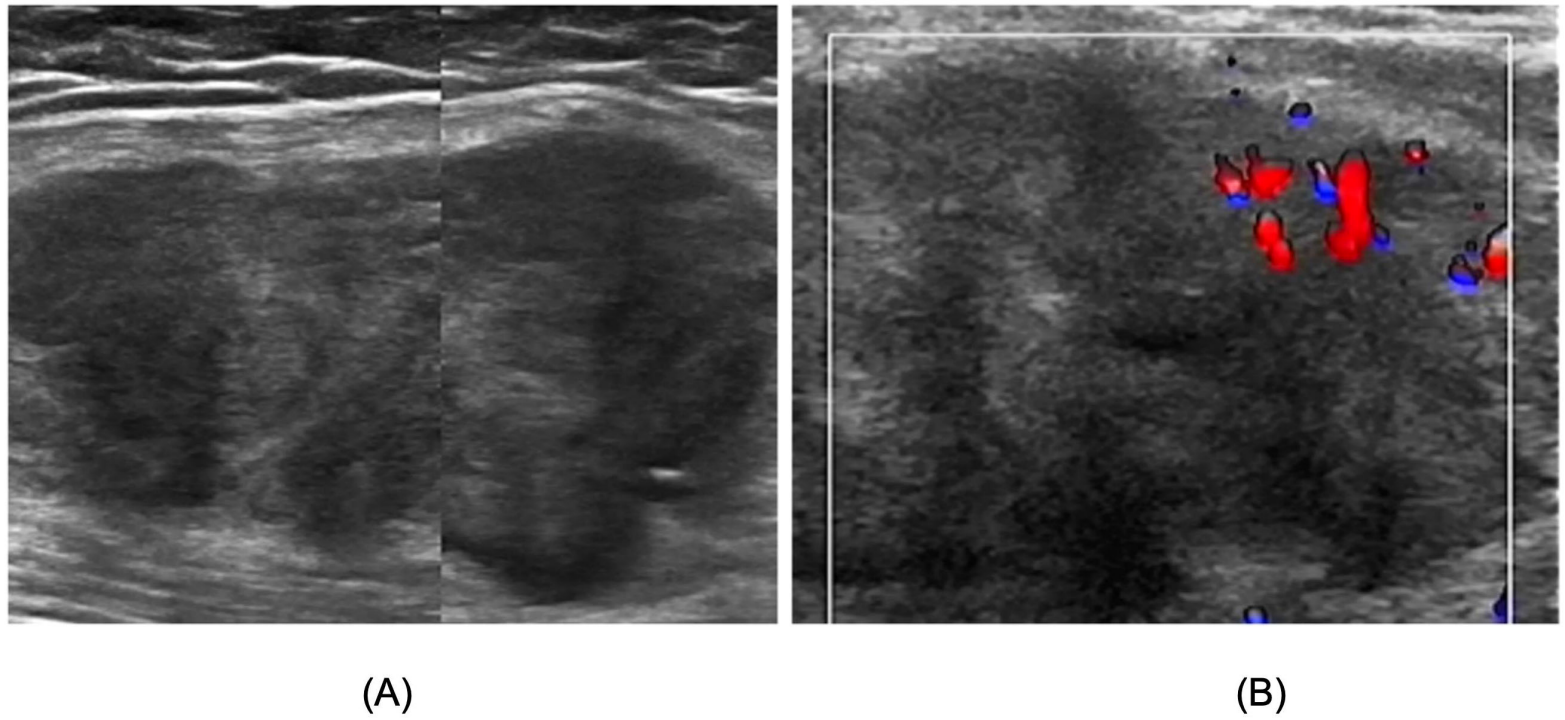
Lower Extremity US image of right thigh skeletal muscle tumor. **(A)** The tumor appears as an irregular hypoechoic mass with indistinct borders from the surrounding muscles, measured approximately 9.4 x 6.6 × 2.7 cm. **(B)** CDFI demonstrated Adler grade 3 blood flow.

**Figure 2:**
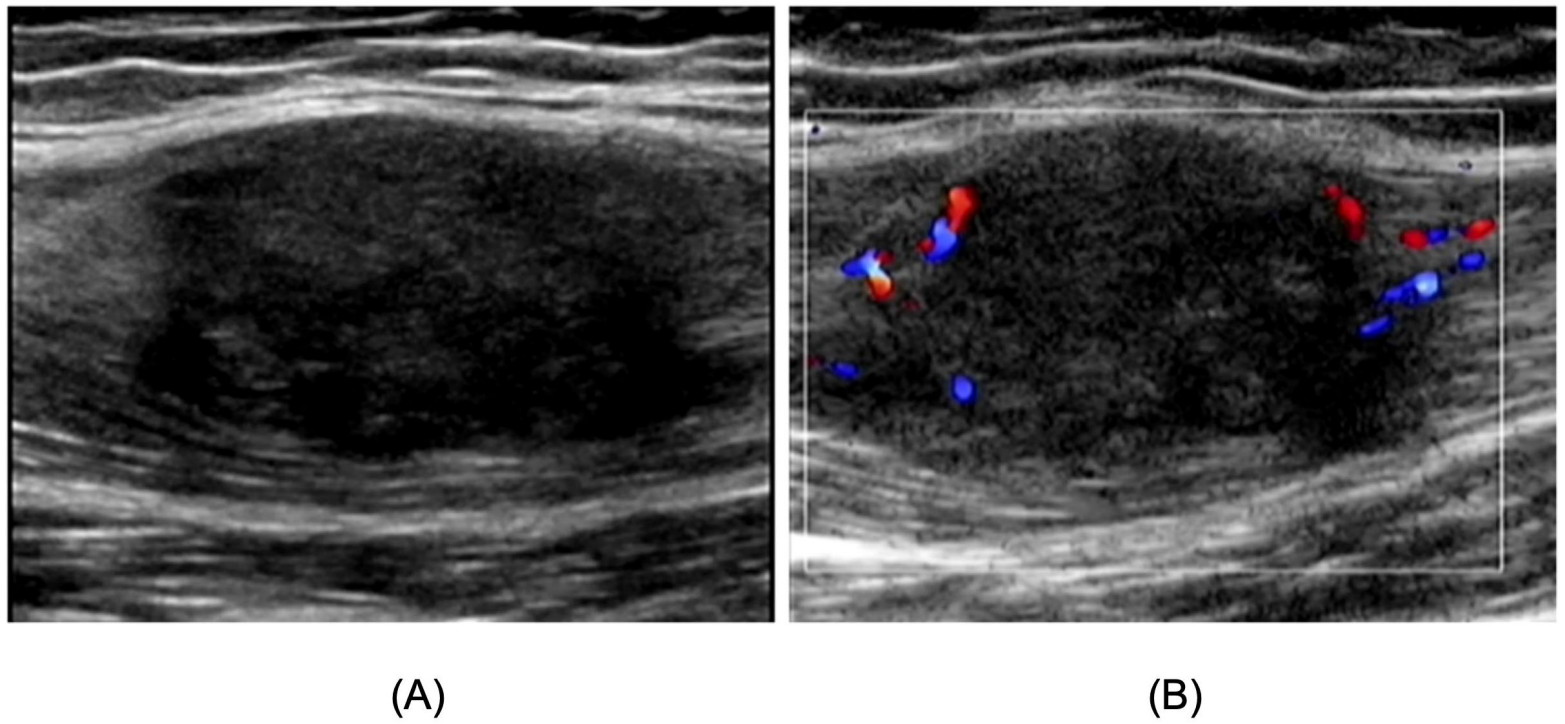
Lower Extremity US image of left thigh skeletal muscle tumor. **(A)** The tumor appears as an irregular hypoechoic mass with indistinct borders from the surrounding muscles, measured approximately 3.4 x 3.0 x 2.6 cm. **(B)** CDFI demonstrated Adler grade 3 blood flow.

**Figure 3:**
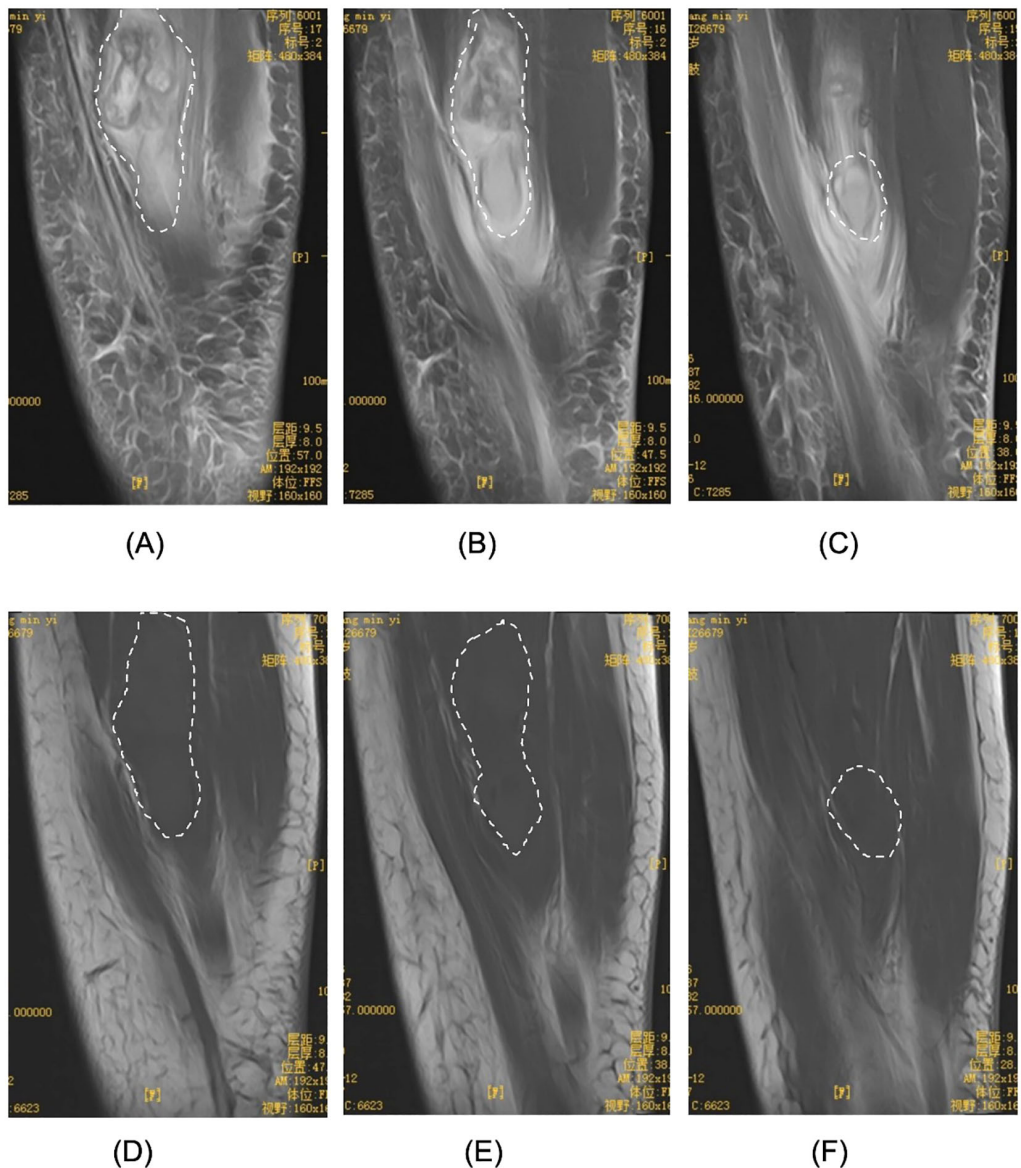
Lower Extremity MRI image of a skeletal muscle tumor in the right thigh. **(A-C)** on fat-suppressed sequences, they appear hyperintense with thick-walled cystic components and indistinct margin, measuring approximately 2.8 × 3.2 × 9.7 cm. **(D-F)** T1-weighted images show mildly hypointense signals with irregular contours.

To further clarify the lesion characteristics, Lower Extremity US revealed irregular hypoechoic masses within the medial thigh muscle groups bilaterally, with indistinct borders from surrounding muscles and heterogeneous internal echo patterns. The right-sided lesion measured approximately 9.4 x 6.6 × 2.7 cm (**Figure 1A**), whereas the left-sided lesion measured approximately 3.4 × 3.0 x 2.6 cm (**Figure 2A**). In both masses, Color Doppler Flow Imaging(CDFI) demonstrated Adler grade 3 blood flow (**Figure 1B**, **2B**), indicating space-occupying lesions. Based on these findings, Lower Extremity MRI was subsequently performed on the right thigh, demonstrating a heterogeneous mass within the medial soft tissues. The lesion was predominantly slightly hypointense on T1-weighted images and hyperintense on fat-suppressed sequences, with an irregular contour, internal thick-walled cystic components, and indistinct margins. The lesion measured approximately 2.8 × 3.2 × 9.7 cm (**Figure 3**), leading to an initial radiologic impression of abscess formation. Lower Extremity MRI of the left thigh was not performed.

**Figure 4:**
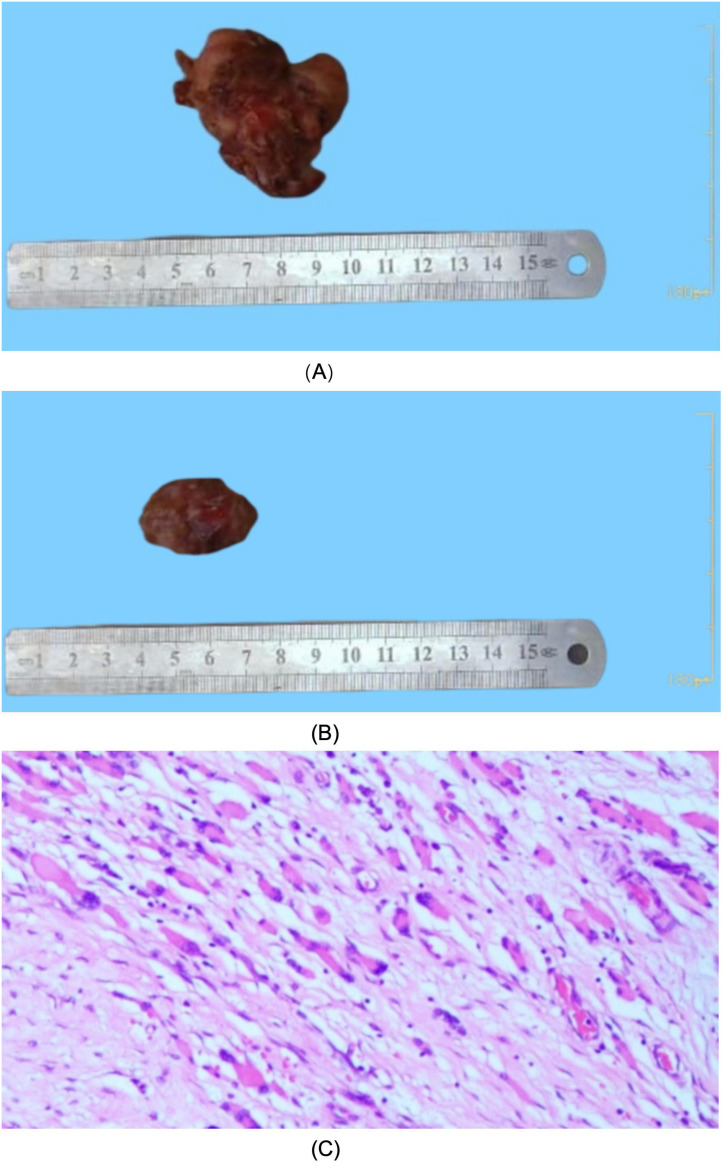
Specimens of bilateral thigh tumors and their HE-stained images. **(A)** Specimen of skeletal muscle tumor from the right thigh, measuring approximately 5.0 × 4.0× 3.0 cm. **(B)** Specimen of skeletal muscle tumor from the left thigh, measuring approximately 3.0 x 2.0 x 2.0 cm. **(C)** Microscopic appearance showed neoplastic tissue with infiltration by tumor cells which are round to oval shape with nuclear pleomorphism, irregular nuclear membrane, and moderate cytoplasm(100x).

Given the imaging findings, laboratory evaluation was conducted and revealed a markedly elevated C-reactive protein level (approximately 92 mg/L), whereas other biochemical parameters were within normal limits. The patient subsequently underwent surgical excision of the bilateral thigh masses for definitive diagnosis and treatment. Intraoperatively, an irregular, firm solid mass measuring approximately 5.0 × 4.0 × 3.0 cm with poorly defined borders was identified within the medial muscle group of the right thigh (**Figure 4A**). In the left thigh, a solid, ovoid mass measuring approximately 3.0 x 2.0 x 2.0 cm with intermediate firmness was found in the mid-belly of the rectus femoris muscle (**Figure 4B**). Histopathological examination demonstrated malignant tumors arising from skeletal muscle in both thighs, Microscopic appearance showed neoplastic tissue with infiltration by tumor cells which are round to oval shape with nuclear pleomorphism, irregular nuclear membrane, and moderate cytoplasm (**Figure 4C**). Immunohistochemical analysis showed positivity for CK, CD31, CD34, and focal ERG, loss of INI-1 expression, and negativity for SMA, ALK, desmin, MUC4, S-100, and TLE1. The Ki-67 proliferation index was 30%. Taken together with hematoxylin–eosin morphology, these immunophenotypic findings confirmed a diagnosis of classic-type ES.

## Discussion

ES is an exceedingly rare soft-tissue sarcoma, accounting for approximately 1% of all soft-tissue sarcomas. It predominantly affects young individuals, with a male-to-female ratio of about 2:1, and its precise etiology remains unclear (6, 7). ES most commonly arises in the extremities and only rarely involves the axial skeleton. Owing to its tendency to mimic chronic inflammatory conditions in clinical presentation, timely and accurate diagnosis is often challenging (7, 8). According to the 2020 World Health Organization classification of soft-tissue and bone tumors, ES is categorized into two principal subtypes: the classic (distal) type and the proximal type (9).

However, there are significant differences in the clinical and pathological characteristics between the two subtypes of ES. The classic type of ES typically occurs in young men and presents as subcutaneous or deep dermal nodules in the distal extremities. It is characterized by a nodular growth pattern (10) and a propensity for local recurrence, regional lymph node involvement, and distant metastases to the lungs, brain, and bone (3, 11). In contrast, the proximal type more frequently arises along the midline of the trunk and in the perineal and genital regions. These tumors are generally larger, with diameters that may reach up to 20 cm, display more aggressive biological behavior, and are associated with a poorer prognosis (12). Importantly, the distinction between classic and proximal types i primarily on histopathological features rather than anatomical location alone. Clinically, ES usually manifests as a slowly enlarging, firm, palpable mass and may occasionally be accompanied by overlying skin ulceration (13). Consequently, accurate and timely diagnosis is essential, as delayed recognition is closely associated with suboptimal treatment and unfavorable clinical outcomes (12). Case reports published over the past five years are shown in **Table 1**.

**Table 1:** A literature review of case reports published in the past five years.

Age and gender	Tumor location/size	Imaging findings	Treatment strategy	Prognosis	References
37 years old man with NF1	Right thigh/14x11.5x10cm	MRI heterogeneous solid-cystic signal, predominantly showing signs of haemorrhage, and demonstrated heterogeneous enhancement	Palliative marginal resection	Died on postoperative day 6	(6)
42 years old man	Lumbar spine/no size	MRI showed heterogeneous signal intensity and fracture of L4 vertebral body; Contrast MRI showed homogenous enhancement	Radiotherapy and chemotherapy were planned	Died 4 weeks postoperative due to multiorgan dysfunction	(7)
54 years old man	Cervicothoracic spine/1.3× 0.7x0.8cm	MRI demonstrated a with heterogeneous and infiltrative, poorly defined margins	Extensive resection and radiotherapy	Died within five months from presentation.	(12)
42 years old man with NF1	Posterior neck/3.5×3.5cm	PET and enhanced CT showed subcutaneous nodular lesions with enhancement	Excision of the suspicious lesions, radiotherapy with chemotherapy	No recurrence	(13)
30 years old man	Lumbar spine/7.5 × 4.5 × 3.5cm	MRI showed whole L1 vertebral body with low signal on the T1-WI and high signal intensity on the fat-suppressed T2-WI, uniform enhancement; PET showed an abnormal uptake in the tumor	Chemotherapy, total en bloc resection	Died one year after diagnosis	(10)
82 years old woman	Left adrenal/6.5×5.1×5.0cm	CT/MRI merely describes the size	Nephrectomy, with the adrenalectomy and mass excision	At four months postoperative, the patient is doing well	(4)
23 years old woman	Anterior tongue/1.1×1.5 ×1.3cm	Enhanced CT revealed a well-defined and peripherally enhancing lesion	Surgical resection, radiotherapy	18-month post-radiation therapy showed no evidence of local, regional or distant disease	(8)

Abbreviations: NF1, neurofibromatosis type I; T1-W1, T1-weighted image; T2-W1, T2-weighted image; MRI, magnetic resonance; Imaging; PET, positron emission tomography; CT, computed tomography.

Against this clinical and pathological background, the present case is noteworthy for the simultaneous involvement of bilateral thigh skeletal muscles, an exceedingly rare presentation that has been seldom reported. This unusual pattern suggests that ES may possess a biological propensity for multifocal occurrence or early occult dissemination. Although histopathological examination remains the diagnostic gold standard, imaging modalities such as US and MRI play an important complementary role in clinical evaluation. However, ES lacks specific imaging hallmarks. On MRI, ES typically demonstrates iso- to slightly hypointense signal intensity on T1-weighted images and iso- to slightly hyperintense signal intensity on T2-weighted images, with heterogeneous post-contrast enhancement and poorly defined margins (14). These features substantially overlap with those of inflammatory lesions, abscesses, and other soft-tissue tumors, thereby complicating accurate radiologic diagnosis.

Consistent with these diagnostic challenges, in the present case the Lower Extremity MRI appearance of the right-sided lesion closely resembled that of an abscess, and the markedly elevated C-reactive protein level further increased the likelihood of misdiagnosis. This finding underscores the importance of considering malignant entities such as ES, even when imaging features are suggestive of an inflammatory process. Nevertheless, despite its tendency to mimic inflammation on MRI, this modality remains highly valuable for delineating the relationship between the tumor and adjacent muscle compartments, thereby enabling accurate preoperative localization, assessment of local disease extent, and postoperative surveillance.

Similarly, US provides complementary information in the evaluation of ES. On US, ES typically appears as a solid mass within the subcutaneous tissue or muscle, characterized by an irregular contour, ill-defined margins, heterogeneous echotexture, frequent areas of liquefaction, occasional calcification, and relatively increased vascularity; however, these features are largely nonspecific. In the present case, the bilateral lesions also exhibited atypical ultrasonographic findings, complicating differentiation from benign entities such as schwannoma or nodular fasciitis. Schwannomas are described as well-circumscribed masses with smooth, regular margins, displacing local structures, usually without invading surrounding tissues and normal nerve fibers can be seen entering and exiting the mass (15). Nodular fasciitis is typically displayed as a hypoechoic solid mass with various margins including irregular and angular, which can be suspected as carcinoma (16). The typical US appearance of an abscess is characterized by an ill-defined mass with heterogeneous internal echoes, potentially surrounded by a hyperechoic inflammatory capsule. CDFI reveals no blood flow signals within the lesion, though increased blood flow may be observed in the surrounding tissues. Although ES’s US findings are difficult to differentiate from benign conditions, US remains valuable for lesion localization, assessment of tumor extent, and postoperative follow-up. Notably, de Visscher and colleagues have proposed that contemporary minimally invasive diagnostic strategies—combining ultrasonographic assessment with sentinel lymph node biopsy of the regional nodal basin—should be incorporated into the preoperative staging of patients with ES (17). Taken together, the extreme rarity of ES, its nonspecific imaging manifestations, and limited clinical awareness likely contributed to the initial diagnostic difficulty encountered in this case.

Once the histopathological diagnosis of ES is confirmed, standard management primarily involves radical surgical resection, which may include amputation (18). When regional lymph node involvement or distant metastasis is suspected, sentinel lymph node biopsy is recommended, and adjuvant radiotherapy should be considered in cases with suspected or confirmed metastatic disease (13). In contrast, ES is largely refractory to chemotherapy, and currently available chemotherapeutic regimens have shown limited therapeutic benefit (11). Recent clinical trials identified the *EZH2* inhibitor tazemetostat as a promising treatment for ES, with 15% of patients showing a response (reduction in tumor size) (19). Prognosis in ES is influenced by multiple factors, including patient age, tumor location, histological grade, TNM stage, and treatment strategy. Patients with localized disease have a reported 5-year survival rate of approximately 75% and a 10-year survival rate of about 46% following surgical resection (20). Overall, younger patients and those with the classic subtype tend to experience more favorable outcomes, whereas proximal-type ES is associated with a poorer prognosis (6). In the present case, the patient was referred to a tertiary center after surgery; however, further follow-up data were unavailable, which represents a limitation of this report.

In summary, ES is an aggressive malignant neoplasm characterized by repeated soft-tissue recurrences and eventual metastasis to the lungs, brain, and bone. Radical surgical resection remains the cornerstone of treatment; however, long-term outcomes are often unsatisfactory. Because a definitive diagnosis cannot be reliably established on imaging alone, accurate identification remains challenging. Preoperative US can delineate tumor size, location, its relationship to the skin layers, and regional lymph node involvement, thereby supporting differential diagnosis and surgical planning, whereas postoperative US is useful for surveillance of tumor recurrence. MRI further contributes to preoperative localization, assessment of local tumor extent, and postoperative follow-up. Although the clinical application of US in musculoskeletal disorders has expanded in recent years, ES-specific reports remain limited. Greater familiarity with the clinical and imaging characteristics of ES may therefore improve diagnostic accuracy. Nevertheless, definitive diagnosis ultimately relies on histopathological evaluation, particularly immunohistochemical analysis.

## Data Availability

The original contributions presented in the study are included in the article/supplementary material. Further inquiries can be directed to the corresponding authors.
